# Differential Expression of miRNAs in Hypoxia (“HypoxamiRs”) in Three Canine High-Grade Glioma Cell Lines

**DOI:** 10.3389/fvets.2020.00104

**Published:** 2020-02-28

**Authors:** Jennifer Koehler, Maninder Sandey, Nripesh Prasad, Shawn A. Levy, Xiaozhu Wang, Xu Wang

**Affiliations:** ^1^Department of Pathobiology, Auburn University, Auburn, AL, United States; ^2^HudsonAlpha Institute for Biotechnology, Huntsville, AL, United States; ^3^Alabama Agricultural Experimental Station, Auburn University, Auburn, AL, United States

**Keywords:** canine, dog, glioma, glioblastoma, hypoxia, microRNA, miRNA, cancer

## Abstract

Dogs with spontaneous high-grade gliomas increasingly are being proposed as useful large animal pre-clinical models for the human disease. Hypoxia is a critical microenvironmental condition that is common in both canine and human high-grade gliomas and drives increased angiogenesis, chemo- and radioresistance, and acquisition of a stem-like phenotype. Some of this effect is mediated by the hypoxia-induced expression of microRNAs, small (~22 nucleotides long), non-coding RNAs that can modulate gene expression through interference with mRNA translation. Using an *in vitro* model with three canine high-grade glioma cell lines (J3T, SDT3G, and G06A) exposed to 72 h of 1.5% oxygen vs. standard 20% oxygen, we examined the global “hypoxamiR” profile using small RNA-Seq and performed pathway analysis for targeted genes using both Panther and NetworkAnalyst. Important pathways include many that are well-established as being important in glioma biology, general cancer biology, hypoxia, angiogenesis, immunology, and stem-ness, among others. This work provides the first examination of the effect of hypoxia on miRNA expression in the context of canine glioma, and highlights important similarities with the human disease.

## Introduction

Glioblastomas are malignant primary brain tumors that comprise ~1/5 of all primary brain tumors in adult humans and are associated with a rapid clinical decline and short survival time after diagnosis, with or without treatment (1). Canine patients also suffer from malignant gliomas, with tumors that are largely similar in histopathological appearance, clinical progression, and some key molecular features (2–5). Dogs are increasingly of interest in comparative glioma research as potential animal models, owing to the fact that gliomas develop in these patients in the context of a large, complex brain, an intact immune system, increased genomic similarity to humans as compared to rodent models, and similar environmental exposures (6–11). Because the pathology classification of canine gliomas has recently undergone a revision as part of the efforts of the Comparative Brain Tumor Consortium in the Comparative Oncology Program of the National Institute of Health's National Cancer Institute (6, 12), in discussions here, the canine tumors will be referred to by the broader appellation “high-grade gliomas,” while the human tumors will retain their more specific designations when available and appropriate. Like many solid tumors, gliomas *in vivo* exhibit considerable phenotypic heterogeneity related to both acquired mutation-induced genetic heterogeneity and methylation patterns, as well as regional phenotypic differences secondary to microenvironmental influences such as oxygen level, pH, and extracellular matrix stiffness (13–17). The role of the microenvironment, especially hypoxia, is of significant interest because of the profound impact that it can have on tumor progression, the success of targeted therapeutic modalities, and the expression level of biomarkers. Hypoxia secondary to ineffective tumor vascularization and tissue infarction is common in both human and canine high-grade gliomas, and is an extremely important driver of tumor growth, invasiveness, chemo- and radioresistance, and acquisition of a stem-like phenotype (18–22).

When cells are exposed to hypoxic stress, they temporarily arrest in the cell cycle, decrease their energy consumption, secrete survival factors, and increase expression of proangiogenic genes (23). Transcriptional, translational, and post-translational mechanisms contribute to these responses, and the primary modulators of gene regulation in response to hypoxia are the hypoxia-inducible factors (HIFs). The HIFs are members of the basic helix-loop-helix/Per-Arnt-Sim (bHLH/PAS) family of transcription factors that function as heterodimers composed of an oxygen-labile α subunit and a constitutively-expressed β subunit. Mammalian species possess three α isoforms: HIF1α, HIF2α, and HIF3α. HIF1α and, to a lesser extent, HIF2α, are the best characterized and most structurally similar, while HIF3α's function is less clear as it exists as multiple splice variants, some of which exert inhibitory transcriptional control over the other isoforms (24). In normoxic conditions, HIF proteins have a short half-life of <5 min (25, 26), being targeted for ubiquitination and proteasomal degradation by the von Hippel-Lindau tumor suppressor protein after hydroxylation of specific proline residues within an oxygen-dependent domain by HIFα-specific prolyl hydroxylases (PHDs) (27–29). Under hypoxic conditions, oxygen is unavailable to PHDs as a co-substrate, and HIFα subunits are stabilized and subsequently translocate to the nucleus where they form heterodimers with the constitutively expressed β subunits. These heterodimers then bind to hypoxia-response elements (HREs) of the genome containing a core RCGTG sequence, as well as to HIF ancillary sequences composed of imperfect tandem repeats that recruit transcription factor complexes other than HIF as well (30). More than 70 genes have been identified as bona fide direct HIF targets containing an HRE, and more than 200 genes have been identified using microarray as being affected by hypoxia and therefore direct or indirect targets of HIFs (31).

MicroRNAs (miRNAs, miRs) are a class of small (~22 nucleotides long), non-coding RNAs that can modulate gene expression through interference with mRNA translation (32). They are key factors in the regulation of gene expression in both normal and neoplastic tissue, and they are being actively explored as prognostic indicators, biomarkers, and diagnostic and therapeutic targets (33–35). Many miRs are highly conserved across species, especially within the so-called “seed” region that serves as the main determinant of target specificity (36). Hypoxia is a potent regulator of miRNA expression (reviewed in (37, 38)), and miRNAs in turn are potent regulators of many aspects of glioblastoma behavior (39–41). The hypoxic miRNA signatures (designated by the authors as “hypoxamiRs”) of the human glioblastoma cell line U87 in experimental hypoxia, in conjunction with a limited number of patient-derived tumors, were profiled by Agrawal et al. using small RNA-Seq, with identification of several hypoxamiRs that promote hypoxic survival and chemoresistance (42). One of the most interesting identified miRNAs in this study was miR-210-3p (100% sequence similarity with canine miRNA-210), which targets HIF3α, the previously mentioned negative regulator of the important oxygen-labile HIFα subunits 1 and 2. To date there has been no published work examining the miRNA profile of canine high-grade gliomas in response to hypoxia.

In the present study, we evaluated the effect of hypoxia on the miRNA expression profile of the canine high-grade glioma cell lines J3T, SDT3G, and G06A using small RNA-Seq. The J3T line was derived from a tumor originally classified as an anaplastic astrocytoma, in a 10-year-old male Boston Terrier (43). The SDT3G and G06A lines were derived from tumors originally classified as glioblastomas, in a 12-year-old male Bulldog (SDT3G) and a 2-year-old female ovariohysterectomized Australian Shepherd (G06A) (44). In a study examining hierarchical clustering of specimens based on chromosomal aberrations, the J3T and G06A cells clustered most closely with other tumors classified morphologically as glioblastoma, while the SDT3G cells clustered most closely with tumors classified morphologically as anaplastic oligodendroglioma (high grade oligodendroglioma) and an oligoastrocytoma (undefined glioma) (3). None of these cell lines is commercially available, and were generously offered by the original investigators, Drs. Michael Berens (J3T) and Dr. Peter Dickinson (SDT3G, G06A).

## Materials and Methods

### Cell Lines

All canine glioma cell lines were cultured in standard two-dimensional culture in media consisting of Dulbecco's Modified Eagle Medium with high glucose and glutamine, supplemented with 10% heat-inactivated fetal calf serum and 100 units/mL of penicillin, 100 μg/mL of streptomycin, and 0.25 μg/mL of amphotericin B. Following transfer to our laboratory from the laboratory of the original investigators, cells were initially expanded and aliquots of 1 x 10^6^ cells were frozen at −180°C to create a stock of early-passage cells. Routine testing for mycoplasma using various commercially available kits on initial and subsequent random aliquots of cells was negative, and cells were confirmed to be purely of canine origin using previously published multiplex PCR primers (45). Cells were used in assays within ten passages of this initial passage. “Normoxic” cells were incubated under standard conditions of 37°C, 20% oxygen, 5% CO_2_, and 100% humidity. “Hypoxic” cells were incubated in conditions described below.

### Hypoxic Conditions

Hypoxic conditions were produced using a proprietary commercial hypoxia chamber with dual-gas controller (C-chamber with Pro-Ox gas controller, BioSpherix Ltd., Parish, NY, USA). Briefly, this system consists of a self-contained fully adjustable and continuously monitored and regulated chamber that sits within a standard CO_2_ incubator. “Hypoxic” conditions in these experiments consisted of an atmosphere that was 1.5% oxygen, 5% CO_2_, and 100% humidity, while “normoxic” conditions consisted of 20% oxygen, 5% CO_2_, and 100% humidity.

### RNA Extraction

**Figure 1:**
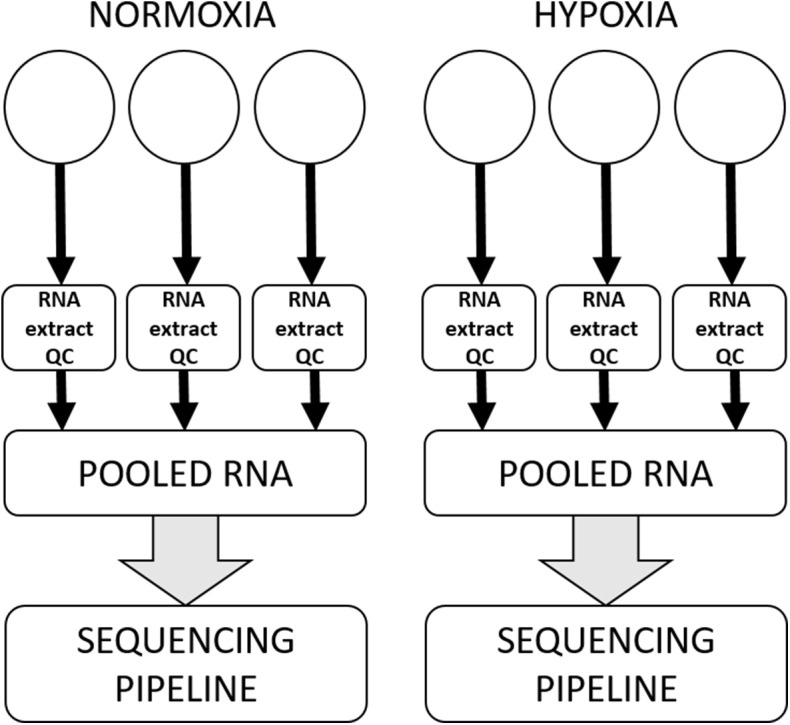
Workflow for a single cell line in a single experiment. All cell lines were subjected to three separate experiments on three separate days, within which there were three technical replicates for each condition. RNA was extracted and subjected to quality control processes separately from each technical replicate, and then technical replicates were pooled to enter the sequencing pipeline.

All canine high-grade glioma cell lines were seeded at previously optimized density in three wells of a tissue culture-treated rigid six-well polystyrene plate and allowed to attach and rest for approximately 12–18 h prior to having media refreshed and being incubated in experimental oxygen concentrations for 72 h. Each experiment was performed three separate times, with samples in technical triplicate for each condition in each experiment. RNA from each of the replicates was extracted using a commercial column-based kit (Qiagen miRNANeasy with on-column RNase-free DNase treatment) and stored at −80°C until shipment on dry ice to HudsonAlpha Institute for Biotechnology Genomic Services Laboratory (HAIB-GSL). At HAIB-GSL, RNA was quality assurance tested using an Agilent Bioanalyzer to generate an RNA integrity number (RIN). Samples that passed all minimum quality testing benchmarks as set by HAIB-GSL were put into the sequencing pipeline, with technical replicates pooled prior to entering the sequencing pipeline (Figure 1).

### Sequencing

Total RNA from each sample was taken into a small RNA library preparation protocol using a NEBNext Small RNA Library Prep Set for Illumina (New England BioLabs Inc., Ipswich, MA, USA) according to the manufacturer's protocol. Briefly, 3‘ adapters were ligated to total input RNA followed by hybridization of multiplex SR RT primers and ligation of multiplex 5‘ SR adapters. Reverse transcription (RT) was performed using ProtoScript II RT for 1 h at 50°C. Immediately after the RT reaction, PCR amplification was performed for 15 cycles using LongAmp Taq 2X master mix. Illumina indexed primers were added to uniquely barcode each sample. Post-PCR material was purified using a QIAquick PCR purification kit (Qiagen Inc., Valencia, CA, USA). Post-PCR yield and concentration of the prepared libraries were assessed using a Qubit 2.0 Fluorometer (Invitrogen, Carlsbad, California, USA) and DNA 1000 chip on an Agilent 2100 Bioanalyzer (Applied Biosystems, Carlsbad, CA, USA), respectively. Size selection of small RNA was done using a 3% dye free agarose gel cassette on a Pippin prep instrument (Sage Science Inc., Beverly, MA, USA). Post-size selection yield and concentration of the libraries were assessed using Qubit 2.0 Fluorometer and DNA High sensitivity chip on Agilent 2100 Bioanalyzer, respectively. Accurate quantification for sequencing applications was performed using the qPCR-based KAPA Biosystems Library Quantification kit (Kapa Biosystems, Inc., Woburn, MA, USA). Each library was diluted to a final concentration of 1.25 nM and pooled in equimolar ratios prior to clustering. Single End (SE) sequencing (50 bp) was performed to generate at least 15 million reads per sample on an Illumina HiSeq2500 sequencer (Illumina, Inc., San Diego, CA, USA).

Post-processing of the sequencing reads from small RNA-Seq experiments from each sample was performed as per the HAIB-GSL unique in-house pipeline. Briefly, quality control checks on raw sequence data from each sample was performed using FastQC (Babraham Bioinformatics, London, UK). Raw reads were imported on a commercial data analysis platform AvadisNGS (Strand Scientifics, CA, USA). Adapter trimming was done to remove ligated adapter from 3' ends of the sequenced reads with only one mismatch allowed, poorly aligned 3' ends were also trimmed. Sequences shorter than 15 nucleotides length were excluded from further analysis. Trimmed Reads with low qualities (base quality score <30, alignment score <95, mapping quality <40) were also removed. Filtered reads were then used to extract and count the small RNA which was annotated with miRNAs from the miRNA database miRDB (46). The quantification operation carries out measurement at both the gene level and at the active region level. Active region quantification considers only reads whose 5' end matches the 5' end of the mature miRNA annotation. Samples were then grouped as identifiers and the differential expression of each miRNA was calculated on the basis of their fold change observed between different groups (hypoxia vs. normoxia), with a 1.5-fold threshold. Canine miRNA sequences were evaluated for homology with specific human miRNAs (Supplemental Table 1), then those homologous miRNA sequences were input into miRDB (46, 47) to look for predicted gene targets with a target score of ≥80%. Initial pathway analysis was conducted by inputting these predicted gene targets into the Panther database version 14.1 (www.pantherdb.org)(48) Additional pathway enrichment analysis was conducted with this same set of target genes using NetworkAnalyst 3.0, which utilizes KEGG pathways in its analysis (49). The adjusted *P* < 0.05 was used as a cutoff for the significantly enriched pathways in NetworkAnalyst.

## Results

**Figure 2:**
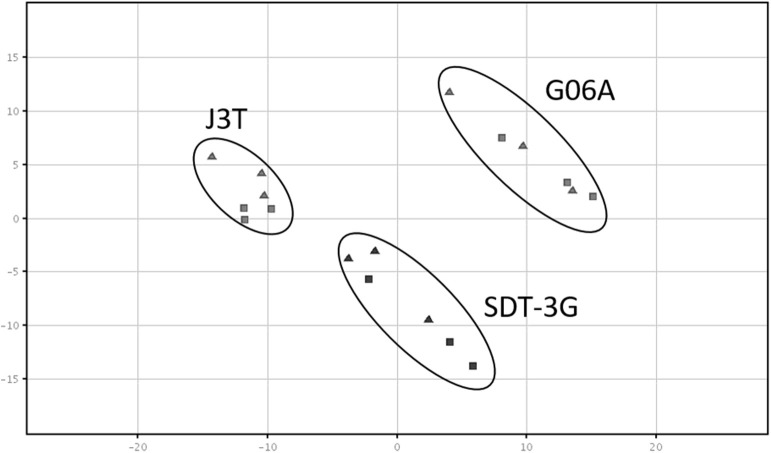
Principal component analysis plot of samples. All sample types cluster separately, with a closer relationship of cell line samples (normoxia and hypoxia) with each other. The triangles indicate normoxic samples, while squares indicate hypoxic samples.

**Figure 3:**
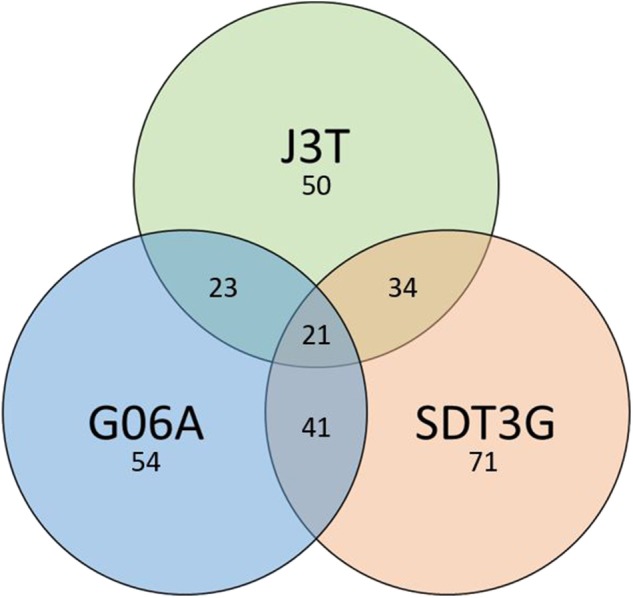
Venn diagram of numbers of unique and overlapping differentially expressed miRNAs in hypoxia vs. normoxia for all cell lines.

**Figure 4:**
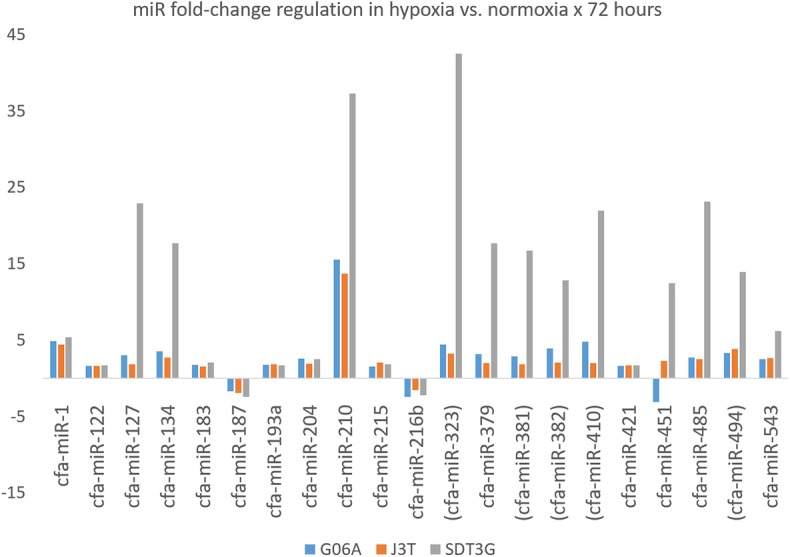
Fold-change regulation for miRNAs differentially regulated in all three cell lines. Parentheses indicate members of the miR-154 family.

Principle component analysis of samples indicated that they clustered separately, with the hypoxic and normoxic samples of a given cell line clustering closer to each other than between cell lines (Figure 2). When comparing miRNA expression in hypoxia compared to normoxia, the J3T, SDT3G, and G06A cell lines all had both unique and overlapping differentially expressed (1.5-fold up or down) miRNAs (Figure 3). Of these differentially regulated miRNAs, J3T had a total of 128, G06A had 139, and SDT3G had 167. Ninety eight miRNAs were differentially regulated in at least two of the three cell lines, and 21 miRNAs were differentially regulated in all three cell lines (Figures 3, 4).

When examining the target gene set from the 21 miRNAs differentially expressed in all three cell lines using the Panther tool, the highest number of hits were in the Wnt signaling pathway, followed by the pathways listed in Table 1 (minimum of 10 hits) and in Supplemental Table 2 (all hits), many of which are known to be involved in cancer biology, progression, metastasis, or acquisition or maintenance of a stem-like phenotype. Because there is some degree of bias inherent in any pathway-prediction database, predicted gene targets for miRNAs that were differentially upregulated in all three cell lines were also analyzed for involvement in specific pathways using NetworkAnalyst, including miR-1-3p, miR-122-5p, miR-134-5p, miR-183-5p, miR-193a-5p, miR-204-5p, miR-210-3p, miR-215-5p, miR-216b-5p, miR-323a-3p, miR-379-5p, miR-381-3p, miR-382-3p, miR-410-3p, miR-421, miR-451a, miR-485-5p, miR-494-3p, and miR 543. Target genes of miR-127-3p and miR-187-3p were excluded, as the number of total target genes was fewer than ten. Many target genes are involved in cancer-related pathways, and many others are involved in pathways that share genes with developmental or cancer-related pathways (Supplemental Table 3).

**Table 1:** miRNA-targeted gene pathway analysis using Panther with a minimum of 10 hits.

**Wnt signaling pathway (P00057)**	**16**
Apoptosis signaling pathway (P00006)	14
FGF signaling pathway (P00021)	14
Gonadotropin-releasing hormone receptor pathway (P06664)	14
Huntington disease (P00029)	14
Inflammation mediated by chemokine and cytokine signaling pathway (P00031)	14
Muscarinic acetylcholine receptor 1 and 3 signaling pathway (P00042)	14
P53 pathway feedback loops 1 and 2 (P04392)	14
Alzheimer disease-presenilin pathway (P00004)	13
Angiogenesis (P00005)	13
Axon guidance mediated by semaphorins (P00007)	13
EGF receptor signaling pathway (P00018)	13
TGF-beta signaling pathway (P00052)	13
Heterotrimeric G-protein signaling pathway-Gi alpha and Gs alpha mediated pathway (P00026)	12
Integrin signaling pathway (P00034)	12
PDGF signaling pathway (P00047)	12
Beta2 adrenergic receptor signaling pathway (P04378)	11
CCKR signaling map (P06959)	11
Ionotropic glutamate receptor pathway (P00037)	11
Metabotropic glutamate receptor group III pathway (P00039)	11
p53 pathway (P00059)	11
VEGF signaling pathway (P00056)	11
5HT2 type receptor mediated signaling pathway (P04374)	10
Cadherin signaling pathway (P00012)	10
Heterotrimeric G-protein signaling pathway-Gq alpha and Go alpha mediated pathway (P00027)	10
Insulin/IGF pathway-protein kinase B signaling cascade (P00033)	10
Oxytocin receptor mediated signaling pathway (P04391)	10
p38 MAPK pathway (P05918)	10
Parkinson disease (P00049)	10
Ras Pathway (P04393)	10
Thyrotropin-releasing hormone receptor signaling pathway (P04394)	10
Ubiquitin proteasome pathway (P00060)	10

In particular, some target genes of certain miRNAs are significantly enriched in some pathways (adjusted *P* < 0.05, Table 2). The target genes of miR-204-5p are significantly enriched in longevity regulating pathway; the target genes of miR-323a-3p are significantly enriched in gap junction; the target genes of miR-382-3p are significantly enriched in oocyte meiosis, tight junction, progesterone-mediated oocyte maturation and hippo signaling pathway; the target genes of miR-485-5p are significantly enriched in longevity regulating pathway (similar to miR-204-5p); the target genes of miR 543 are significantly enriched in osteoclast differentiation, salmonella infection, cGMP-PKG signaling pathway, circadian entrainment, choline metabolism in cancer and vasopressin-regulated water reabsorption. It is important to bear in mind that although enrichment of some pathways as determined by statistical analysis is of interest, less-frequently involved pathways may still be of significant biological relevance. Additionally, pathways listed as developmental or degenerative (ex. “oocyte meiosis”) may share genes with cancer-related pathways.

**Table 2:** Significantly enriched pathways of target genes from the identified miRNAs using NetworkAnalyst with *p* < 0.05.

**microRNA name**	**Enriched pathway**	**FDR (adjusted *P*-value)**
miRNA-204-5p	Longevity regulating pathway	0.0164
miRNA-323a-3p	Gap junction	0.0160
miRNA-382-3p	Oocyte meiosis	0.0262
	Tight junction	0.0363
	Progesterone-mediated oocyte maturation	0.0363
	Hippo signaling pathway	0.0363
miRNA-485-5p	Longevity regulating pathway	0.0095
miRNA 543	Osteoclast differentiation	0.0481
	Salmonella infection	0.0481
	cGMP-PKG signaling pathway	0.0481
	Circadian entrainment	0.0481
	Choline metabolism in cancer	0.0481
	Vasopressin-regulated water reabsorption	0.0481

## Discussion

Because a comprehensive review of every differentially regulated miRNA and pathway in this data set is beyond the scope of a single manuscript, for the purposes of discussion we have chosen to present a brief overview of some of the most highly differentially regulated miRNAs and those that may be relevant from a comparative cancer biology or therapeutic targeting perspective. Because so little canine-specific miRNA experimental data exists in the literature, information about these selected miRNAs in dogs, if available, is therefore presented along with available information on human glioma, general cancer biology, developmental physiology, and/or hypoxia-related physiology. Of the miRNAs upregulated in one or more canine cell lines in hypoxia, many have been associated with differential regulation in human cancer cell lines in hypoxia, human glioblastomas as compared to normal brain, or human tumors with poorer clinical outcomes, and many have had hypoxia response elements identified within their promoters.

### miR-210: Upregulated in Hypoxia in Canine High-Grade Glioma Cell Lines

miR-210 is well-recognized as being highly upregulated in hypoxia in a variety of solid tumors including glioblastomas (42, 50–58), a finding which has been further confirmed mechanistically by both the identification of a hypoxia response element (HRE) in its promoter (42) and the close correlation of miR-210 expression with vascular endothelial growth factor (*VEGF*) expression (53). In human glioblastoma cells, increased miR-210 expression leads to decreased apoptosis and increased cell proliferation (58). When comparing human patient-derived glioblastoma samples with normal brain, miR-210 expression is highly overexpressed in the tumors, and expression is highly correlated with both hypoxia markers *VEGF* and carbonic anhydrase 9 (*CA9*) (58). In addition to glioblastomas, miR-210 overexpression has been documented in breast, lung, head and neck, and pancreatic carcinomas (55, 59, 60), and is associated with poor outcomes as determined by disease-free survival, progression-free survival, and relapse-free survival in a variety of cancers including glioblastoma (61–65). In one examination of miRNAs in a dataset of 480 human glioblastomas included in The Cancer Genome Atlas (TCGA), low levels of miRs 210, 155, 329, and 323 in tumors were associated with longer overall survival of these patients (65). In terms of its utility in diagnostic pathology, miR-210 is markedly overexpressed in human glioblastomas when compared to oligodendrogliomas, primarily due to a more frequent incidence of promoter methylation in the latter (66). Whether this will be recapitulated in the context of the extensively necrotic high-grade oligodendrogliomas that are much more common in canine patients than human patients deserves further investigation.

Angiogenesis is robust in high-grade gliomas, and both human and canine tumors are characterized by proliferations of microvasculature that is florid but also paradoxically ineffective. Experimentally, miR-210 overexpression in human umbilical vein endothelial cells (HUVECs) in normoxia enhances both angiogenesis and VEGF-driven cell migration (67). In non-neoplastic tissues within the context of brain injury, miR-210 expression levels are increased in brain tissue after ischemia (68). Beyond reinforcing the importance of this miRNA in hypoxia, this also raises concerns for tumor-tissue crosstalk in glioma *in vivo*, since exosome-mediated transfer of miR-210 from glioblastoma cells to neighboring cells has been documented (69). In addition to the previously mentioned target of HIF3α, other identified miR-210 target genes are myriad and are involved in a wide variety of biological processes and pathways including but not limited to hypoxia, angiogenesis, proliferation, apoptosis, neurogenesis, cell differentiation, and complex tumor type-dependent roles as both an oncogene and tumor suppressor (70, 71).

**Figure 5:**
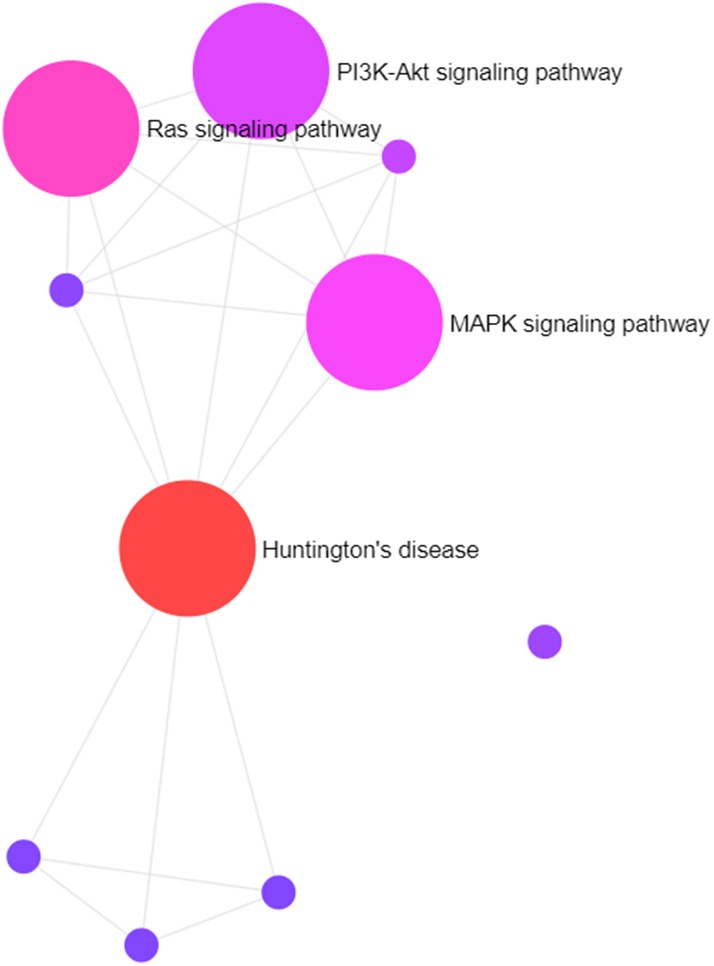
Visual representation of primary pathways targeted by miR-210. The size of the circles correlates roughly with the proportion of genes that are involved in a particular pathway.

Gene targets of miR-210 in our data set are involved in the Huntington's disease, Ras signaling, MAPK signaling, PI3K-Akt signaling, and IGF signaling pathways, among others (Figure 5). The last four are well-established important cancer pathways. The Huntington's pathway, while less intuitive based on the name, contains many cancer-relevant genes including those involved in oxidative phosphorylation, movement of brain-derived neurotrophic factor by molecular motors, glutamate receptor binding, p53 signaling, and CREB binding. Additionally, there is an intriguingly low cancer incidence in human patients with Huntington's disease and a body of work examining the role of mutant and wild-type huntingtin protein in cancer development and progression (46). Marked upregulation of this “master” hypoxamiR in these three canine high-grade glioma cell lines serves as confirmation of experimental hypoxia induction and also confirms that glioma-bearing canine patients may be useful models for studying therapies directed at this target (72, 73).

### miR-323: Upregulated in Hypoxia in Canine High-Grade Glioma Cell Lines

Like miR-210, miR-323 contains a hypoxia response element within its promoter, and low levels of miR-323 are associated with longer overall survival in human glioblastoma patients (65). Intriguingly, of the 21 miRNAs that are differentially regulated in all three cell lines, five (miR-323, miR-381, miR-382, miR-410, and miR-494) are all members of the miR-154 family. Targets of miR-323 in Panther pathway analysis have the highest number of hits in the integrin signaling and platelet-derived growth factor (PDGF) signaling pathways, followed by ubiquitin proteasome, Wnt, and EGFR. In NetworkAnalyst, there is significant enrichment in gap junction pathways. An important target of miR-323 is the antisense non-coding RNA in the *INK4* locus (*ANRIL*) (74). Single-nucleotide polymorphisms within the tumor suppressor *p16(INK4a)/p14(ARF)* 3' untranslated region are associated with multiple cancers including glioblastoma, and in breast cancer the CG allele is linked to more aggressive tumors with higher levels of ANRIL (75). Within the context of human prostate cancer, miR-323 enhances tumor angiogenesis, and ectopic expression of miR-323 promotes cell proliferation and colony formation *in vitro* and tumor growth in *in vivo* xenograft mouse models (76, 77). Finally, miR-323 is upregulated in ischemia/reperfusion-injured neurons and oxygen-glucose deprived neurons (78), further supporting its role as a hypoxamiR.

### miR-1: Upregulated in Hypoxia in Canine High-Grade Glioma Cell Lines

Within the context of embryonic development, miR-1 is critical for the differentiation of embryonic stem cells into vascular smooth muscle cells (79). A primary target of this miRNA is the Kruppel-like factor 4 transcription factor (*KLF4*), which inhibits epithelial-to-mesenchymal transition through the regulation of E-cadherin gene expression (80). miR-1 appears to have divergent roles as either tumor-suppressive or oncogenic based on tumor type, with some tumors overexpressing and others underexpressing. Regulation is complex, via copy number variation, epigenetic, transcriptional, and post-transcriptional mechanisms (81). In the context of the experimental glioblastoma microenvironment, miR-1 directly targets annexin A2 and the proto-oncogene *MET*, and miR-1-loaded extracellular vesicles lead to diminished invasion and proliferation in targeted cells (82). Overexpression of the miR-1 target *MET* has been documented in canine gliomas (83). miR-1 is markedly downregulated in glioblastomas as compared to normal brain and, despite its interaction with *KLF4*, restoration of miR-1 has been proposed as a therapeutic modality (84). miR-1 is highly downregulated in canine hepatocellular carcinoma as compared to normal liver (85), and in osteosarcoma as compared to normal bone (86). Top Panther targeted pathways for miR-1 in our data set include the GNRH receptor pathway, inflammation mediated by chemokine and cytokine signaling pathway, G-protein coupled receptor signaling, Wnt signaling, as well as CCKR, angiogenesis, and lesser numbers of hits for p53, FGF, EGF, PDGF, and VEGF, among others. Despite the fact that this miRNA is upregulated in hypoxia as compared to normoxia in our model, preliminary work in our lab also shows much lower levels of miR-1 in normoxic canine glioma cell lines as compared to normoxic primary cultured astrocytes (unpublished data).

### miR-134: Upregulated in Hypoxia in Canine High-Grade Glioma Cell Lines

In Panther analysis of our predicted miR-134 target gene set, top pathway hits include the integrin, angiogenesis, inflammation mediated by cytokine/chemokine, and EGFR signaling pathways. miR-134 is overexpressed in glioblastomas as compared to low-grade oligodendrogliomas in people, a finding which is not related to differences in copy number (66). Much like the overexpression of miR-210, the validation of this finding in canine high-grade oligodendrogliomas, the vast majority of which contain areas of extensive necrosis and its attendant hypoxic microenvironment, deserves further investigation; the oligodendroglial tumors in this human study by definition lack significant necrosis. In a single study, miR-134 has been reported as being overexpressed in human high-grade glioma cell lines and tumors as compared to cultured astrocytes, but experimental investigation of the effect of *in vitro* hypoxia was not part of this work. In this same study, stimulation of the epidermal growth factor receptor (EGFR) and platelet-derived growth factor receptor (PDGFR) significantly repressed miR-134 levels (87); in other words, there appeared to be an inverse relationship between these growth factors and miR-134. Yet, in other studies in human glioblastoma cell lines, hypoxia caused increased expression of *EGFR* via HIF2α (88) and upregulation of miR-134 expression (42). A positive correlation between miR-134 expression and matrix metalloproteinase-9 (*MMP-9*) exists in esophageal cancer, and high expression in tumors is associated with shorter survival time (89). *MMP-9* is known to be overexpressed in gliomas, with higher expression associated with higher grade (90), and it can serve as both a biomarker and a predictor of survival in patients (91, 92). Increased levels of MMP-9 have been documented in the cerebrospinal fluid of dogs with intracranial tumors including histologically confirmed gliomas (93, 94).

### miR-494: Upregulated in Hypoxia in Canine High-Grade Glioma Cell Lines

miR-494 is overexpressed in glioblastomas as compared to normal brain, and is a statistically important miRNA with regard to survival when patients are segregated according to therapy vs. no-therapy (95). Target genes for this miRNA reported in the literature include those involved in cell signaling, metabolism, and apoptosis; increased expression of this miRNA leads to increased cell proliferation and decreased apoptosis of glioblastoma cell lines *in vitro* (95). miR-494 enhances invasiveness of gliomas via *EGFR* upregulation, protein kinase B (Akt) activation, and extracellular signal-regulated kinase (ERK) activation, and downregulation of this miRNA in glioblastoma stem-like cells leads to increased apoptosis and suppresses invasion and proliferation (95). For this reason, it has been proposed as a therapeutic target in human glioblastoma (96, 97)

### miR-216b: Downregulated in Hypoxia in Canine High-Grade Glioma Cell Lines

miR-216b is significantly downregulated in glioma cells and tissues in comparison to normal brain, and ectopic expression of this miRNA inhibits proliferation and invasion in both *in vitro* and *in vivo* xenograft models (98). In this same study, the authors identify forkhead box protein M1 (*FOXM1*) as a direct target, and ectopic expression of miR-216b led to a decrease in FOXM1 protein levels and percentage of Ki-67-positive cells in xenograft models. A similar mechanism has been shown for melanoma (99), hepatocellular carcinoma (100), non-small-cell lung carcinoma (101), and osteosarcoma (102), and low miR-216b levels in tumors are independent poor prognostic indicators in many of these cancers. In pancreatic cancer, this miRNA functions in a tumor-suppressive way by targeting translationally controlled tumor protein (*TPT1*), and levels of this miRNA are significantly associated with large tumor size and advanced TNM stage (103). In breast cancer, the oncogene histone deacetylase 8 (*HDAC8*), which accelerates proliferation and progression, is targeted by miR-216-5p via binding to the 3'UTR (104). Taken together, there is strong evidence to suggest that downregulation of this miRNA leads to proliferation and invasion in a wide variety of cancers, including glioma.

In order to understand how dogs with spontaneously occurring high-grade gliomas can best be used to study targeted therapies that may of benefit to patients of both species, it will be important to explore the similarities and differences that are inherent in this and any other comparative model. Microenvironmental conditions, especially hypoxia, are common in high-grade gliomas of both species and drive the development of many genotypic, phenotypic, and epigenetic traits that conspire to thwart existing therapies. For that reason, hypoxia-modulated pathways are tempting therapeutic targets. The fact that canine tumors often have extensive necrosis (associated with hypoxic or even anoxic environments) like their human counterparts supports their use in helping to unravel the tangled web of molecular interactions fueled by low oxygen levels. Here we present the first published work examining the experimental hypoxamiR landscape in canine high-grade glioma. Pathway analysis of targeted genes in this model highlights the importance of the Wnt pathway, and confirms the importance of hypoxia in many other pathways known to be involved in general cancer as well as glioma-specific oncogenesis, progression, metastasis, and promotion of a treatment-resistant stem-like phenotype. Taken together, the data offer further support for the use of dogs with spontaneous high-grade gliomas as a useful comparative model. In order to translate these *in vitro* results into clinically relevant findings, future work should focus on evaluation of miRNA expression in three-dimensional culture systems in serum-free media and in canine tumors *in vivo*, as there are limitations associated with standard cell culture practices and their effect on gene expression.

## Data Availability Statement

The miRNA sequencing data has been uploaded and can be accessed from NCBI Sequence Read Archive (SRA) under BioprojectID PRJNA605029 (https://www.ncbi.nlm.nih.gov/bioproject/605029).

## Author Contributions

Experiments were performed by JK (cell culture), MS (RNA extraction), NP (sequencing), and SL (sequencing). Data analysis was performed by NP, XiW, and XuW. The manuscript was written by JK, with methods and results input from NP, XiW, and XuW.

### Conflict of Interest

The authors declare that the research was conducted in the absence of any commercial or financial relationships that could be construed as a potential conflict of interest.

## References

[1] Ostrom Q Gittleman H Farah P Ondracek A Chen Y Wolinsky Y. CBTRUS statistical report: primary brain and central nervous system tumors diagnosed in the United States 2006-2010. Neuro Oncol. (2013) 15 i1–ii56. 10.1093/neuonc/not151 24137015 PMC3798196

[2] Boudreau CE York D Higgins RJ LeCouteur RA Dickinson PJ. Molecular signalling pathways in canine gliomas. Vet Comp Oncol. (2017) 15:133–50. 10.1111/vco.12147 25808605

[3] Dickinson PJ York D Higgins RJ LeCouteur RA Joshi N Bannasch D. Chromosomal aberrations in canine gliomas define candidate genes and common pathways in dogs and humans. J Neuropathol Exp Neurol. (2016) 75:700–10. 10.1093/jnen/nlw042 27251041 PMC4913437

[4] Higgins RJ Dickinson PJ LeCouteur RA Bollen AW Wang H Wang H. Spontaneous canine gliomas: overexpression of EGFR, PDGFRalpha and IGFBP2 demonstrated by tissue microarray immunophenotyping. J Neurooncol. (2010) 98:49–55. 10.1007/s11060-009-0072-5 19967449

[5] York D Sproul CD Chikere N Dickinson PJ Angelastro JM. Expression and targeting of transcription factor ATF5 in dog gliomas. Vet Comp Oncol. (2018) 16:102–7. 10.1111/vco.12317 28480569 PMC5677578

[6] LeBlanc AK Mazcko C Brown DE Koehler JW Miller AD Miller CR. Creation of an NCI comparative brain tumor consortium: informing the translation of new knowledge from canine to human brain tumor patients. Neuro Oncol. (2016) 18:1209–18. 10.1093/neuonc/now051 27179361 PMC4999002

[7] Dickinson PJ LeCouteur RA Higgins RJ Bringas JR Larson RF Yamashita Y. Canine spontaneous glioma: a translational model system for convection-enhanced delivery. Neuro Oncol. (2010) 12:928–40. 10.1093/neuonc/noq046 20488958 PMC2940703

[8] Herranz C Fernandez F Martin-Ibanez R Blasco E Crespo E De la Fuente C. Spontaneously arising canine glioma as a potential model for human glioma. J Comp Pathol. (2016) 154:169–79. 10.1016/j.jcpa.2015.12.001 26804204

[9] Khanna C Lindblad-Toh K Vail D London C Bergman P Barber L. The dog as a cancer model. Nat Biotechnol. (2006) 24:1065–6. 10.1038/nbt0906-1065b 16964204

[10] Paoloni M Khanna C. Translation of new cancer treatments from pet dogs to humans. Nat Rev Cancer. (2008) 8:147–56. 10.1038/nrc2273 18202698

[11] Rowell JL McCarthy DO Alvarez CE. Dog models of naturally occurring cancer. Trends Mol Med. (2011) 17:380–8. 10.1016/j.molmed.2011.02.004 21439907 PMC3130881

[12] Koehler JW Miller AD Miller CR Porter B Aldape K Beck J. A revised diagnostic classification of canine glioma: towards validation of the canine glioma patient as a naturally occurring preclinical model for human glioma. J Neuropathol Exp Neurol. (2018) 77:1039–54. 10.1093/jnen/nly085 30239918 PMC6181180

[13] Edwards LA Woo J Huxham LA Verreault M Dragowska WH Chiu G. Suppression of VEGF secretion and changes in glioblastoma multiforme microenvironment by inhibition of integrin-linked kinase (ILK). Mol Cancer Ther. (2008) 7:59–70. 10.1158/1535-7163.MCT-07-0329 18202010

[14] Heddleston JM Li Z McLendon RE Hjelmeland AB Rich JN. The hypoxic microenvironment maintains glioblastoma stem cells and promotes reprogramming towards a cancer stem cell phenotype. Cell Cycle. (2009) 8:3274–84. 10.4161/cc.8.20.9701 19770585 PMC2825672

[15] Hoelzinger DB Demuth T Berens ME. Autocrine factors that sustain glioma invasion and paracrine biology in the brain microenvironment. J Natl Cancer Inst. (2007) 99:1583–93. 10.1093/jnci/djm187 17971532

[16] Oliver L Olivier C Marhuenda FB Campone M Vallette FM. Hypoxia and the malignant glioma microenvironment: regulation and implications for therapy. Curr Mol Pharmacol. (2009) 2:263–84. 10.2174/1874467210902030263 20021464

[17] Goldbrunner RH Bernstein JJ Tonn JC. Cell-extracellular matrix interaction in glioma invasion. Acta Neurochir. (1999) 141:295–305. 10.1007/s007010050301 10214487

[18] Evans SM Jenkins KW Chen HI Jenkins WT Judy KD Hwang WT. The relationship among hypoxia, proliferation, and outcome in patients with *de novo* glioblastoma: a pilot study. Transl Oncol. (2010) 3:160–9. 10.1593/tlo.09265 20563257 PMC2887645

[19] Lund EL Hog A Olsen MW Hansen LT Engelholm SA Kristjansen PE. Differential regulation of VEGF, HIF1alpha and angiopoietin-1,−2 and−4 by hypoxia and ionizing radiation in human glioblastoma. International journal of cancer. J Int Cancer. (2004) 108:833–8. 10.1002/ijc.11662 14712484

[20] Qiang L Wu T Zhang HW Lu N Hu R Wang YJ. HIF-1alpha is critical for hypoxia-mediated maintenance of glioblastoma stem cells by activating Notch signaling pathway. Cell Death Differ. (2012) 19:284–94. 10.1038/cdd.2011.95 21818118 PMC3263503

[21] Seidel S Garvalov BK Wirta V von Stechow L Schanzer A Meletis K. A hypoxic niche regulates glioblastoma stem cells through hypoxia inducible factor 2 alpha. Brain. (2010) 133:983–95. 10.1093/brain/awq042 20375133

[22] Yang L Lin C Wang L Guo H Wang X. Hypoxia and hypoxia-inducible factors in glioblastoma multiforme progression and therapeutic implications. Exp Cell Res. (2012) 318:2417–26. 10.1016/j.yexcr.2012.07.017 22906859

[23] Majmundar AJ Wong WJ Simon MC. Hypoxia-inducible factors and the response to hypoxic stress. Mol Cell. (2010) 40:294–309. 10.1016/j.molcel.2010.09.022 20965423 PMC3143508

[24] Gu YZ Moran SM Hogenesch JB Wartman L Bradfield CA. Molecular characterization and chromosomal localization of a third alpha-class hypoxia inducible factor subunit, HIF3alpha. Gene Exp. (1998) 7:205–13. 9840812 PMC6151950

[25] Huang LE Arany Z Livingston DM Bunn HF. Activation of hypoxia-inducible transcription factor depends primarily upon redox-sensitive stabilization of its alpha subunit. J Biol Chem. (1996) 271:32253–9. 10.1074/jbc.271.50.32253 8943284

[26] Yu AY Frid MG Shimoda LA Wiener CM Stenmark K Semenza GL. Temporal, spatial, and oxygen-regulated expression of hypoxia-inducible factor-1 in the lung. Am J Physiol. (1998) 275:L818–26. 10.1152/ajplung.1998.275.4.L818 9755115

[27] Kaelin WG Jr. The von Hippel-Lindau tumour suppressor protein: O2 sensing and cancer. Nat Rev Cancer. (2008) 8:865–73. 10.1038/nrc2502 18923434

[28] Kaelin WG Jr Ratcliffe PJ. Oxygen sensing by metazoans: the central role of the HIF hydroxylase pathway. Mol Cell. (2008) 30:393–402. 10.1016/j.molcel.2008.04.009 18498744

[29] Maxwell PH Wiesener MS Chang GW Clifford SC Vaux EC Cockman ME. The tumour suppressor protein VHL targets hypoxia-inducible factors for oxygen-dependent proteolysis. Nature. (1999) 399:271–5. 10.1038/20459 10353251

[30] Kimura H Weisz A Ogura T Hitomi Y Kurashima Y Hashimoto K. Identification of hypoxia-inducible factor 1 ancillary sequence and its function in vascular endothelial growth factor gene induction by hypoxia and nitric oxide. J Biol Chem. (2001) 276:2292–8. 10.1074/jbc.M008398200 11056166

[31] Elvidge GP Glenny L Appelhoff RJ Ratcliffe PJ Ragoussis J Gleadle JM. Concordant regulation of gene expression by hypoxia and 2-oxoglutarate-dependent dioxygenase inhibition: the role of HIF-1alpha, HIF-2alpha, and other pathways. J Biol Chem. (2006) 281:15215–26. 10.1074/jbc.M511408200 16565084

[32] Bartel DP. MicroRNAs: genomics, biogenesis, mechanism, and function. Cell. (2004) 116:281–97. 10.1016/S0092-8674(04)00045-5 14744438

[33] Calin GA Croce CM. MicroRNA signatures in human cancers. Nat Rev Cancer. (2006) 6:857–66. 10.1038/nrc1997 17060945

[34] Farazi TA Spitzer JI Morozov P Tuschl T. miRNAs in human cancer. J Pathol. (2011) 223:102–15. 10.1002/path.2806 21125669 PMC3069496

[35] Garzon R Fabbri M Cimmino A Calin GA Croce CM. MicroRNA expression and function in cancer. Trends Mol Med. (2006) 12:580–7. 10.1016/j.molmed.2006.10.006 17071139

[36] Warnefors M Liechti A Halbert J Valloton D Kaessmann H. Conserved microRNA editing in mammalian evolution, development and disease. Genome Biol. (2014) 15:R83. 10.1186/gb-2014-15-6-r83 24964909 PMC4197820

[37] Loscalzo J. The cellular response to hypoxia: tuning the system with microRNAs. J Clin Invest. (2010) 120:3815–7. 10.1172/JCI45105 20972325 PMC2965005

[38] Nallamshetty S Chan SY Loscalzo J. Hypoxia: a master regulator of microRNA biogenesis and activity. Free Radic Biol Med. (2013) 64:20–30. 10.1016/j.freeradbiomed.2013.05.022 23712003 PMC3762925

[39] Brower JV Clark PA Lyon W Kuo JS MicroRNAs in cancer: glioblastoma and glioblastoma cancer stem cells. Neurochem Int. (2014) 77C:68–77. 10.1016/j.neuint.2014.06.002 PMC4390175 24937770

[40] Henriksen M Johnsen KB Andersen HH Pilgaard L Duroux M MicroRNA expression signatures determine prognosis and survival in glioblastoma multiforme-a systematic overview. Mol Neurobiol. (2014) 50:896–913. 10.1007/s12035-014-8668-y 24619503 PMC4225053

[41] Li R Gao K Luo H Wang X Shi Y Dong Q. Identification of intrinsic subtype-specific prognostic microRNAs in primary glioblastoma. J Exp Clin Cancer Res. (2014) 33:9. 10.1186/1756-9966-33-9 24438238 PMC3917617

[42] Agrawal R Pandey P Jha P Dwivedi V Sarkar C Kulshreshtha R. Hypoxic signature of microRNAs in glioblastoma: insights from small RNA deep sequencing. BMC Genomics. (2014) 15:686. 10.1186/1471-2164-15-686 25129238 PMC4148931

[43] Berens ME Bjotvedt G Levesque DC Rief MD Shapiro JR Coons SW. Tumorigenic, invasive, karyotypic, and immunocytochemical characteristics of clonal cell lines derived from a spontaneous canine anaplastic astrocytoma. In Vitro Cell Dev Biol Anim. (1993) 29A:310–18. 10.1007/BF02633959 8320182

[44] York D Higgins RJ Lecouteur RA Wolfe AN Grahn R Olby N TP53 mutations in canine brain tumors. Vet Pathol. (2012) 45:796–801. 10.1177/0300985811424734 22002975

[45] Parodi B Aresu O Bini D Lorenzini R Schena F Visconti P. Species identification and confirmation of human and animal cell lines: a PCR-based method. Biotechniques. (2002) 32:432–4. 10.2144/02322rr05 11848419

[46] Wong N Wang X. miRDB: an online resource for microRNA target prediction and functional annotations. Nucleic Acids Res. (2015) 43:D146–52. 10.1093/nar/gku1104 25378301 PMC4383922

[47] Liu W Wang X. Prediction of functional microRNA targets by integrative modeling of microRNA binding and target expression data. Genome Biol. (2019) 20:18. 10.1186/s13059-019-1629-z 30670076 PMC6341724

[48] Mi H Muruganujan A Ebert D Huang X Thomas PD. PANTHER version 14: more genomes, a new PANTHER GO-slim and improvements in enrichment analysis tools. Nucleic Acids Res. (2019) 47:D419–26. 10.1093/nar/gky1038 30407594 PMC6323939

[49] Zhou G Soufan O Ewald J Hancock REW Basu N Xia J. NetworkAnalyst 3.0: a visual analytics platform for comprehensive gene expression profiling and meta-analysis. Nucleic Acids Res. (2019) 47:W234–41. 10.1093/nar/gkz240 30931480 PMC6602507

[50] Dang K Myers KA. The role of hypoxia-induced miR-210 in cancer progression. Int J Mol Sci. (2015) 16:6353–72. 10.3390/ijms16036353 25809609 PMC4394536

[51] Devlin C Greco S Martelli F Ivan M. miR-210: more than a silent player in hypoxia. IUBMB Life. (2011) 63:94–100. 10.1002/iub.427 21360638 PMC4497508

[52] Gee HE Camps C Buffa FM Patiar S Winter SC Betts G. hsa-mir-210 is a marker of tumor hypoxia and a prognostic factor in head and neck cancer. Cancer. (2010) 116:2148–58. 10.1002/cncr.25009 20187102

[53] Huang X Le QT Giaccia AJ. MiR-210–micromanager of the hypoxia pathway. Trends Mol Med. (2010) 16:230–7. 10.1016/j.molmed.2010.03.004 20434954 PMC3408219

[54] Noman MZ Janji B Berchem G Chouaib S. miR-210 and hypoxic microvesicles: two critical components of hypoxia involved in the regulation of killer cells function. Cancer Lett. (2016) 380:257–62. 10.1016/j.canlet.2015.10.026 26523672

[55] Qin Q Furong W Baosheng L. Multiple functions of hypoxia-regulated miR-210 in cancer. J Exp Clin Cancer Res. (2014) 33:50. 10.1186/1756-9966-33-50 24909053 PMC4060094

[56] Lee D Sun S Zhang XQ Zhang PD Ho AS Kiang KM. MicroRNA-210 and endoplasmic reticulum chaperones in the regulation of chemoresistance in glioblastoma. J Cancer. (2015) 6:227–32. 10.7150/jca.10765 25663939 PMC4317757

[57] Rosenberg T Thomassen M Jensen SS Larsen MJ Sorensen KP Hermansen SK. Acute hypoxia induces upregulation of microRNA-210 expression in glioblastoma spheroids. CNS Oncol. (2015) 4:25–35. 10.2217/cns.14.48 25586423 PMC6093025

[58] Zhang S Lai N Liao K Sun J Lin Y. MicroRNA-210 regulates cell proliferation and apoptosis by targeting regulator of differentiation 1 in glioblastoma cells. Folia Neuropathol. (2015) 53:236–44. 10.5114/fn.2015.54424 26443314

[59] Malzkorn B Wolter M Liesenberg F Grzendowski M Stuhler K Meyer HE. Identification and functional characterization of microRNAs involved in the malignant progression of gliomas. Brain Pathol. (2010) 20:539–50. 10.1111/j.1750-3639.2009.00328.x 19775293 PMC8094849

[60] Greither T Grochola LF Udelnow A Lautenschlager C Wurl P Taubert H. Elevated expression of microRNAs 155, 203, 210 and 222 in pancreatic tumors is associated with poorer survival. Int J Cancer. (2010) 126:73–80. 10.1002/ijc.24687 19551852

[61] Camps C Buffa FM Colella S Moore J Sotiriou C Sheldon H. hsa-miR-210 Is induced by hypoxia and is an independent prognostic factor in breast cancer. Clin Cancer Res. (2008) 14:1340–8. 10.1158/1078-0432.CCR-07-1755 18316553

[62] Hong L Yang J Han Y Lu Q Cao J Syed L. High expression of miR-210 predicts poor survival in patients with breast cancer: a meta-analysis. Gene. (2012) 507:135–8. 10.1016/j.gene.2012.07.025 22842193

[63] Rothe F Ignatiadis M Chaboteaux C Haibe-Kains B Kheddoumi N Majjaj S. Global microRNA expression profiling identifies MiR-210 associated with tumor proliferation, invasion and poor clinical outcome in breast cancer. PLoS ONE. (2011) 6:e20980. 10.1371/journal.pone.0020980 21738599 PMC3126805

[64] Wang J Zhao J Shi M Ding Y Sun H Yuan F. Elevated expression of miR-210 predicts poor survival of cancer patients: a systematic review and meta-analysis. PLoS ONE. (2014) 9:e89223. 10.1371/journal.pone.0089223 24586608 PMC3930667

[65] Qiu S Lin S Hu D Feng Y Tan Y Peng Y. Interactions of miR-323/miR-326/miR-329 and miR-130a/miR-155/miR-210 as prognostic indicators for clinical outcome of glioblastoma patients. J Transl Med. (2013) 11:10. 10.1186/1479-5876-11-10 23302469 PMC3551827

[66] Lages E Guttin A El Atifi M Ramus C Ipas H Dupre I. MicroRNA and target protein patterns reveal physiopathological features of glioma subtypes. PLoS ONE. (2011) 6:e20600. 10.1371/journal.pone.0020600 21655185 PMC3105101

[67] Fasanaro P D'Alessandra Y Di Stefano V Melchionna R Romani S Pompilio G. MicroRNA-210 modulates endothelial cell response to hypoxia and inhibits the receptor tyrosine kinase ligand Ephrin-A3. J Biol Chem. (2008) 283:15878–83. 10.1074/jbc.M800731200 18417479 PMC3259646

[68] Lou YL Guo F Liu F Gao FL Zhang PQ Niu X. miR-210 activates notch signaling pathway in angiogenesis induced by cerebral ischemia. Mol Cell Biochem. (2012) 370:45–51. 10.1007/s11010-012-1396-6 22833359

[69] Jung KO Youn H Lee CH Kang KW Chung JK. Visualization of exosome-mediated miR-210 transfer from hypoxic tumor cells. Oncotarget. (2017) 8:9899–910. 10.18632/oncotarget.14247 28038441 PMC5354779

[70] Bavelloni A Ramazzotti G Poli A Piazzi M Focaccia E Blalock W. MiRNA-210: a current overview. Anticancer Res. (2017) 37:6511–21. 10.21873/anticanres.12107 29187425

[71] Fasanaro P Greco S Lorenzi M Pescatori M Brioschi M Kulshreshtha R. An integrated approach for experimental target identification of hypoxia-induced miR-210. J Biol Chem. (2009) 284:35134–43. 10.1074/jbc.M109.052779 19826008 PMC2787374

[72] Ren CX Leng RX Fan YG Pan HF Wu CH Ye DQ. MicroRNA-210 and its theranostic potential. Exp Opin Ther Targets. (2016) 20:1325–38. 10.1080/14728222.2016.1206890 27359286

[73] Gupta A Quijano E Liu Y Bahal R Scanlon SE Song E. Anti-tumor activity of miniPEG-gamma-modified PNAs to inhibit MicroRNA-210 for cancer therapy. Mol Ther Nucleic Acids. (2017) 9:111–19. 10.1016/j.omtn.2017.09.001 29246289 PMC5633812

[74] Lan WG Xu DH Xu C Ding CL Ning FL Zhou YL. Silencing of long non-coding RNA ANRIL inhibits the development of multidrug resistance in gastric cancer cells. Oncol Rep. (2016) 36:263–70. 10.3892/or.2016.4771 27121324

[75] Royds JA Pilbrow AP Ahn A Morrin HR Frampton C Russell IA. The rs11515 polymorphism is more frequent and associated with aggressive breast tumors with increased ANRIL and decreased p16. (INK4a) expression. Front Oncol. (2015) 5:306. 10.3389/fonc.2015.00306 26835415 PMC4720739

[76] Gao Q Zheng J. microRNA-323 upregulation promotes prostate cancer growth and docetaxel resistance by repressing p73. Biomed Pharmacother. (2018) 97:528–34. 10.1016/j.biopha.2017.10.040 29091904

[77] Gao Q Yao X Zheng J. MiR-323 inhibits prostate cancer vascularization through adiponectin receptor. Cell Physiol Biochem. (2015) 36:1491–8. 10.1159/000430313 26160610

[78] Yang L Xiong Y Hu XF Du YH. MicroRNA-323 regulates ischemia/reperfusion injury-induced neuronal cell death by targeting BRI3. Int J Clin Exp Pathol. (2015) 8:10725–33. 26617783 PMC4637598

[79] Jakob P Landmesser U. Role of microRNAs in stem/progenitor cells and cardiovascular repair. Cardiovasc Res. (2012) 93:614–22. 10.1093/cvr/cvr311 22135162

[80] Yori JL Johnson E Zhou G Jain MK Keri RA. Kruppel-like factor 4 inhibits epithelial-to-mesenchymal transition through regulation of E-cadherin gene expression. J Biol Chem. (2010) 285:16854–63. 10.1074/jbc.M110.114546 20356845 PMC2878056

[81] Han C Shen JK Hornicek FJ Kan Q Duan Z. Regulation of microRNA-1 (miR-1) expression in human cancer. Biochim Biophys Acta. (2017) 1860:227–32. 10.1016/j.bbagrm.2016.12.004 27923712

[82] Godlewski J Krichevsky AM Johnson MD Chiocca EA Bronisz A. Belonging to a network–microRNAs, extracellular vesicles, and the glioblastoma microenvironment. Neuro Oncol. (2015) 17:652–62. 10.1093/neuonc/nou292 25301812 PMC4482852

[83] Dickinson PJ Roberts BN Higgins RJ Leutenegger CM Bollen AW Kass PH. Expression of receptor tyrosine kinases VEGFR-1 (FLT-1), VEGFR-2 (KDR), EGFR-1, PDGFRalpha and c-Met in canine primary brain tumours. Vet Comp Oncol. (2006) 4:132–40. 10.1111/j.1476-5829.2006.00101.x 19754810

[84] Wang X Huang X Yang Z Gallego-Perez D Ma J Zhao X. Targeted delivery of tumor suppressor microRNA-1 by transferrin-conjugated lipopolyplex nanoparticles to patient-derived glioblastoma stem cells. Curr Pharm Biotechnol. (2014) 15:839–46. 10.2174/1389201015666141031105234 25374033

[85] Lai YC Ushio N Rahman MM Katanoda Y Ogihara K Naya Y. Aberrant expression of microRNAs and the miR-1/MET pathway in canine hepatocellular carcinoma. Vet Comp Oncol. (2018) 16:288–96. 10.1111/vco.12379 29314614

[86] Leonardo L Laura P Serena BM. miR-1 and miR-133b expression in canine osteosarcoma. Res Vet Sci. (2018) 117:133–7. 10.1016/j.rvsc.2017.12.002 29272721

[87] Zhang Y Kim J Mueller AC Dey B Yang Y Lee DH. Multiple receptor tyrosine kinases converge on microRNA-134 to control KRAS, STAT5B, and glioblastoma. Cell Death Differ. (2014) 21:720–34. 10.1038/cdd.2013.196 24440911 PMC3978301

[88] Franovic A Gunaratnam L Smith K Robert I Patten D Lee S. Translational up-regulation of the EGFR by tumor hypoxia provides a nonmutational explanation for its overexpression in human cancer. Proc Natl Acad Sci USA. (2007) 104:13092–7. 10.1073/pnas.0702387104 17670948 PMC1941796

[89] Klimczak-Bitner AA Kordek R Bitner J Musial J Szemraj J. Expression of MMP9, SERPINE1 and miR-134 as prognostic factors in esophageal cancer. Oncol Lett. (2016) 12:4133–8. 10.3892/ol.2016.5211 27895782 PMC5104243

[90] Xu Y Wang J Xu Y Xiao H Li J Wang Z. Screening critical genes associated with malignant glioma using bioinformatics analysis. Mol Med Rep. (2017) 16:6580–9. 10.3892/mmr.2017.7471 28901452 PMC5865802

[91] Hormigo A Gu B Karimi S Riedel E Panageas KS Edgar MA. YKL-40 and matrix metalloproteinase-9 as potential serum biomarkers for patients with high-grade gliomas. Clin Cancer Res. (2006) 12:5698–704. 10.1158/1078-0432.CCR-06-0181 17020973

[92] Iwamoto FM Hottinger AF Karimi S Riedel E Dantis J Jahdi M. Longitudinal prospective study of matrix metalloproteinase-9 as a serum marker in gliomas. J Neurooncol. (2011) 105:607–12. 10.1007/s11060-011-0628-z 21710351 PMC7398421

[93] Mariani CL Boozer LB Braxton AM Platt SR Vernau KM McDonnell JJ. Evaluation of matrix metalloproteinase-2 and−9 in the cerebrospinal fluid of dogs with intracranial tumors. Am J Vet Res. (2013) 74:122–9. 10.2460/ajvr.74.1.122 23270356

[94] Turba ME Forni M Gandini G Gentilini F. Recruited leukocytes and local synthesis account for increased matrix metalloproteinase-9 activity in cerebrospinal fluid of dogs with central nervous system neoplasm. J Neurooncol. (2007) 81:123–9. 10.1007/s11060-006-9213-2 16826366

[95] Cosset E Petty T Dutoit V Tirefort D Otten-Hernandez P Farinelli L. Human tissue engineering allows the identification of active miRNA regulators of glioblastoma aggressiveness. Biomaterials. (2016) 107:74–87. 10.1016/j.biomaterials.2016.08.009 27614160

[96] Kwak SY Yang JS Kim BY Bae IH Han YH. Ionizing radiation-inducible miR-494 promotes glioma cell invasion through EGFR stabilization by targeting p190B rhoGAP. Biochim Biophys Acta. (2014) 1843:508–16. 10.1016/j.bbamcr.2013.11.021 24316134

[97] Li XT Wang HZ Wu ZW Yang TQ Zhao ZH Chen GL. miR-494-3p regulates cellular proliferation, invasion, migration, and apoptosis by PTEN/AKT signaling in human glioblastoma cells. Cell Mol Neurobiol. (2015) 35:679–87. 10.1007/s10571-015-0163-0 25662849 PMC4477718

[98] Zhang T Ma G Zhang Y Huo H Zhao Y. miR-216b inhibits glioma cell migration and invasion through suppression of FoxM1. Oncol Rep. (2017) 38:1751–9. 10.3892/or.2017.5824 28731180

[99] Sun M Wang X Tu C Wang S Qu J Xiao S. microRNA-216b inhibits cell proliferation and migration in human melanoma by targeting FOXM1 *in vitro* and *in vivo*. Cell Biol Int. (2017) 41:1272–82. 10.1002/cbin.10754 28225180

[100] Zheng WW Zhou J Zhang CH Liu XS. MicroRNA-216b is downregulated in hepatocellular carcinoma and inhibits HepG2 cell growth by targeting Forkhead box protein M1. Eur Rev Med Pharmacol Sci. (2016) 20:2541–50. 27383303

[101] Wang L Wang Y Du X Yao Y Wang L Jia Y. MiR-216b suppresses cell proliferation, migration, invasion, and epithelial-mesenchymal transition by regulating FOXM1 expression in human non-small cell lung cancer. Oncotargets Ther. (2019) 12:2999–3009. 10.2147/OTT.S202523 31114243 PMC6489682

[102] Wang W Guo Z Yu H Fan L. MiR-216b inhibits osteosarcoma cell proliferation, migration, and invasion by targeting Forkhead Box M1. J Cell Biochem. (2019) 120:5435–43. 10.1002/jcb.27822 30302807

[103] You Y Tan J Gong Y Dai H Chen H Xu X. MicroRNA-216b-5p functions as a tumor-suppressive RNA by targeting TPT1 in pancreatic cancer cells. J Cancer. (2017) 8:2854–65. 10.7150/jca.18931 28928875 PMC5604218

[104] Menbari MN Rahimi K Ahmadi A Elyasi A Darvishi N Hosseini V. MiR-216b-5p inhibits cell proliferation in human breast cancer by down-regulating HDAC8 expression. Life Sci. (2019) 237:116945. 10.1016/j.lfs.2019.116945 31605710

