# Supplementary material for: Differential Expression of miRNAs in Hypoxia (“HypoxamiRs”) in Three Canine High-Grade Glioma Cell Lines
Source: Front Vet Sci. 2020 Feb 28;7:104. doi: 10.3389/fvets.2020.00104 (PMC7093022; doi:10.3389/fvets.2020.00104)
Supplement: Supplementary file 1 [file Table_1.DOCX]

| **Canine miR** | **Canine sequence** | **Homologous human miR** | **Human sequence** | **% homology** | **miR "family"** |
| --- | --- | --- | --- | --- | --- |
| cfa-miR-1 | uggaauguaaagaaguaugua | hsa-miR-1-3p | uggaauguaaagaaguauguau | 100% | miR-1 |
| cfa-miR-122 | uggagugugacaaugguguuug | hsa-miR-122-5p | uggagugugacaaugguguuug | 100 | miR-122 |
| cfa-miR-127 | ucggauccgucugagcuuggcu | hsa-miR-127-3p | ucggauccgucugagcuuggcu | 100 | miR-127 |
| cfa-miR-134 | ugugacugguugaccagagggg | hsa-miR-134-5p | ugugacugguugaccagagggg | 100 | miR-134 |
| cfa-miR-183 | uauggcacugguagaauucacu | hsa-miR-183-5p | uauggcacugguagaauucacu | 100 | miR-183 |
| cfa-miR-187 | ucgugucuuguguugcagccgg | hsa-miR-187-3p | ucgugucuuguguugcagccgg | 100 | miR-187 |
| cfa-miR-193a | ugggucuuugcgggcgagauga | hsa-miR-193a-5p | ugggucuuugcgggcgagauga | 100 | miR-193 |
| cfa-miR-204 | uucccuuugucauccuaugccu | hsa-miR-204-5p | uucccuuugucauccuaugccu | 100 | miR-204 |
| cfa-miR-210 | acugugcgugugacagcggcuga | hsa-miR-210-3p | cugugcgugugacagcggcuga | 100 | miR-210 |
| cfa-miR-215 | augaccuacgaauugauagaca | hsa-miR-215-5p | augaccuaugaauugacagac | 90.5 | miR-192 |
| cfa-miR-216b | aaaucucugcaggcaaauguga | hsa-miR-216b-5p | aaaucucugcaggcaaauguga | 100 | miR-216 |
| cfa-miR-323 | cacauuacacggucgaccucu | hsa-miR-323a-3p | cacauuacacggucgaccucu | 100 | miR-154 |
| cfa-miR-379 | ugguagacuauggaacguagg | hsa-miR-379-5p | ugguagacuauggaacguagg | 100 | miR-379 |
| cfa-miR-381 | uauacaagggcaagcucucugu | hsa-miR-381-3p | uauacaagggcaagcucucugu | 100 | miR-154 |
| cfa-miR-382 | aaucauucacggacaacacuuu | hsa-miR-382-3p | aaucauucacggacaacacuu | 100 | miR-154 |
| cfa-miR-410 | aauauaacacagauggccugu | hsa-miR-410-3p | aauauaacacagauggccugu | 100 | miR-154 |
| cfa-miR-421 | aucaacagacauuaauugggcg | hsa-miR-421 | aucaacagacauuaauugggcgc | 100 | miR-95 |
| cfa-miR-451 | aaaccguuaccauuacugaguu | hsa-miR-451a | aaaccguuaccauuacugaguu | 100 | miR-451 |
| cfa-miR-485 | agaggcuggccgugaugaauucg | hsa-miR-485-5p | agaggcuggccgugaugaauuc | 100 | miR-485 |
| cfa-miR-494 | ugaaacauacacgggaaaccuc | hsa-miR-494-3p | ugaaacauacacgggaaaccuc | 100 | miR-154 |
| cfa-miR-543 | aaacauucgcggugcacuucuu | hsa-miR-543 | aaacauucgcggugcacuucuu | 100 | miR-329 |

Supplemental table 1. Homology between canine and human miRNA sequences.
